# Impact of the initial response to COVID‐19 on long‐term care for people with intellectual disability: an interrupted time series analysis of incident reports

**DOI:** 10.1111/jir.12778

**Published:** 2020-09-21

**Authors:** C. Schuengel, J. Tummers, P. J. C. M. Embregts, G. L. Leusink

**Affiliations:** ^1^ Department of Educational and Family Sciences, Amsterdam Public Health Research Institute Vrije Universiteit Amsterdam Amsterdam The Netherlands; ^2^ Department of Information Technology Wageningen University & Research Wageningen The Netherlands; ^3^ Tranzo, Tilburg School of Social and Behavioral Sciences Tilburg University Tilburg The Netherlands; ^4^ Department of Primary and Community Care, Radboud Institute for Health Sciences Radboudumc Nijmegen The Netherlands

**Keywords:** administrative data, aggression, COVID‐19, incident reports, long term care, people with intellectual disability

## Abstract

**Background:**

The lockdown‐measures in response to COVID‐19 taken by long‐term care organisations might have impacted problem behaviour and behavioural functioning of people with intellectual disability. This study tested changes in reported incidents, in particular regarding aggression, unexplained absence and, for contrast, medication errors.

**Methods:**

Metadata on weekly incident and near‐incident reports from 2016 to June 2020 involving over 14 000 clients with mild to serious intellectual disability of 's Heeren Loo, a long‐term care organisation for people with intellectual disability, were subjected to interrupted time series analysis, comparing the COVID‐19 with the pre‐COVID‐19 period.

**Results:**

The imposition of lockdown‐measures coincided with a significant drop in incidents (total, *P* < .001; aggression, *P* = .008; unexplained absences, *P* = .008; and medication errors, *P* < .001). Incidents in total (*P* = .001) and with aggression (*P* < .001) then climbed from this initial low level, while medication errors remained stably low (*P* = .94).

**Conclusion:**

The rise in incidents involving aggression, against the background of generally lowered reporting, underlines the need for pandemic control measures that are suitable for people with intellectual disability in long‐term care.

In response to the spread of SARS‐CoV‐2, governments, public health agencies and care organisations across the world have taken drastic measures to curb the ensuing COVID‐19 pandemic. On top of community interventions for the general public, long‐term care facilities for people with disabilities and elderly people limited visits, activities and supportive services (Salcher‐Konrad *et al*. 2020). These measures were taken to limit the risk of people acquiring infection in aggregate care settings from other clients, visitors or care staff (nosocomial transmission) and followed on early reports of actual outbreaks in nursing homes (e.g. McMichael *et al*. 2020). Daily life was upended for people living within care facilities, adding to concerns among clients and staff about the pandemic and its effects. Effects of these unprecedented actions were highly uncertain, given the limited evidence on changes in behaviour and psychological functioning among people with intellectual disability observed during previous epidemics (Hassiotis *et al*. 2020).

In the Netherlands, the national government ordered on 15 March 2020 that restaurants and bars should be closed, children be kept at home (except for parents working in vital sectors) and distance between people be kept at 1.5 m. Long‐term care organisations for people with intellectual disability across the Netherlands took the decision to reduce their operations to safeguard continuity of basic care. They closed their schools, training and activity centres, shops and restaurants and suspended clinical services and counselling. On 23 March, the government announced a nationwide ‘intelligent lockdown’. This meant that everyone was asked to stay in and work from home, only going out for daily stroll, vital work, caregiving or necessary supplies, while congregations in public spaces of three or more people were banned. No exceptions were made for people in long‐term care facilities. Organisations that operated care home locations for people with disabilities adopted a ‘no visits, unless …’ policy (Vereniging Gehandicaptenzorg Nederland 2020). This policy met with increasing resistance and was replaced on 30 June by policy to allow visits under certain conditions. By then, the first wave of infections had largely abated, and there was less imminent danger for clients and staff.

Routine care data may offer insight into the effects of external events on populations in care, as long as the limitations of such data are taken into account, including their potentially flawed, uncertain, proximate or sparse nature (Wolpert and Rutter 2018). Furthermore, administrative data may be the only way to study events that occur with low frequency without having to overburden clients and staff (Franklin and Thorn 2019). Metadata (describing the source, nature or amount of underlying data) may be especially useful because these data do not refer to individuals and do not invade privacy. Routine care data are collected for the purpose of statistical detection of unwanted trends, such as filing reports of adverse events involving clients and staff, otherwise denoted as ‘incidents’. Incidents may include acts of verbal or physical aggression and violence, unexplained absence, self‐harm, as well as incidents around medication. Reports may also include near‐incidents, where timely interventions prevented the incident from occurring. Incident reports of aggression in particular have shown to be related to clinical risk assessment for aggression (O'Shea *et al*. 2015) and to be a helpful tool for care facilities to take measures towards guarding safety (Malda Castillo *et al*. 2018).

Particularly relevant in the context of the control measures against the pandemic and nosocomial infection are incidents involving aggression and unexplained absences or going missing. During displays of physical or verbal aggression, it may be difficult for coresidents and staff to keep the recommended physical distance (in the Netherlands, 1.5 m). Interventions to prevent or resolve aggressive incidents may also be incompatible with keeping such distance. Clients who have been absent or missing without informing staff of their whereabouts may have had contacts that are difficult to trace, increasing the risk of uncontrolled infection spread. Incidents may also be used to reflect on the impact of changes in rules, routines and social relationships, for example because aggressive incidents are often functionally explained as aversive responses to changes in daily routines, tasks and social interactions (Embregts *et al*. 2009) and may signal people's frustration of their basic needs for autonomy, competence and relatedness (Frielink *et al*. 2018). Unexplained absences may be another response to need frustration, for example as a way to overcome the bans on visiting friends or attachment figures.

While it is plausible that changes in daily life due to COVID‐19 might have increased the risk for incidents, positive reactions may also be possible. These may follow from the suspending of daily obligations such as work, freeing time for leisure activities and from decreasing numbers of people invading the privacy of group homes. It is also possible that the perceived threat of COVID‐19 and clear, strict rules reduced transgressions of rules and boundaries. Therefore, the current study aimed to address the question how incident reports changed in numbers for a large care organisation from before until the end of the initial response phase in which the general public as well as clients of long‐term care were subjected to the most stringent level of measures to control the spread of SARS‐Cov‐2. We specifically examined changes in level and rate of change in incidents of aggression, going missing and unexplained absence. As a contrast, we also tested effects on medication incident reports. Medication errors refer to practices by staff while other incidents involve the behaviour of clients or interactions between clients and staff. Thus, diverging patterns of change from the pre‐COVID‐19 phase to the COVID‐19 phase might aid the interpretation of any changes found.

## Methods

### Design and phases

The study employed a quasi‐experimental interrupted time series design, based on weekly counts of incident reports from 5 September 2016 to 25 June 2020. This period was divided between a pre‐COVID‐19 baseline leading up to the COVID‐19 initial response phase starting on 15 March 2020. On that date, the government announced the start of its strategy of maximum control of the spread of the SARS‐CoV‐2 virus. Simultaneously, long‐term care organisations put day activities, therapies and consultations on hold, paused new admissions and a week later suspended visits to clients at care locations or visits from clients to their families and friends. Thus, 15 March marked a sudden imposition of new invasive rules and routines for people with intellectual disability living in long‐term care facilities.

### Sample and setting

Metadata on the number of incident reports by week were made available by 's Heeren Loo, a large long‐term care organisation for people with intellectual disabilities in the Netherlands that operates about 1000 locations divided across 25 regions. The intellectual disabilities among clients ranged from mild intellectual disability in combination with severe problem behaviour to profound and multiple disability. Ethical approval for the study was granted by the review board of the Faculty of Behavioural and Movement Sciences of Vrije Universiteit Amsterdam (protocol #VCWE‐2020‐128).

### Number of incident reports

Incident reports are part of the mandatory safeguarding and quality system policy of care organisations. Direct care staff are instructed to report incidents and near‐incidents that threatened the safety of clients and staff. This registration enables learning and addressing acute risks. A semi‐structured form asked for descriptions of when and where the incident or near‐incident occurred, who was involved, what type of incident it was, whether people were left injured and how the incident was followed up. Staff could choose to report incidents involving aggression (including verbal and physical aggression, violence and sexual transgressions), fire setting, medication error, unexplained absence or going missing (whereabouts unknown), accidents or other unsafe situations. From July 2019 on, the care organisation implemented a new incident reporting system, keeping however the main incident types the same. This new system was introduced in a staggered fashion over a year across regions in the organisation. The data manager of the care organisation produced from its client data bank a table of week numbers and weekly counts of incidents per type for the analyses. The data manager also provided weekly counts of clients registered with the organisation. These numbers have gradually risen for total number of clients. The period covered by the study started in 2016 when the organisation had 10 877 clients and ended with 14 207 in Week 25 of 2020 (the end of the reported period). However, the number of clients living in a care home location operated by the organisation, who would be involved with the overwhelming majority of incidents, remained constant from 6292 clients living in care homes in 2016 and 6301 clients in 2020.

### Statistical analysis

**Figure 1:**
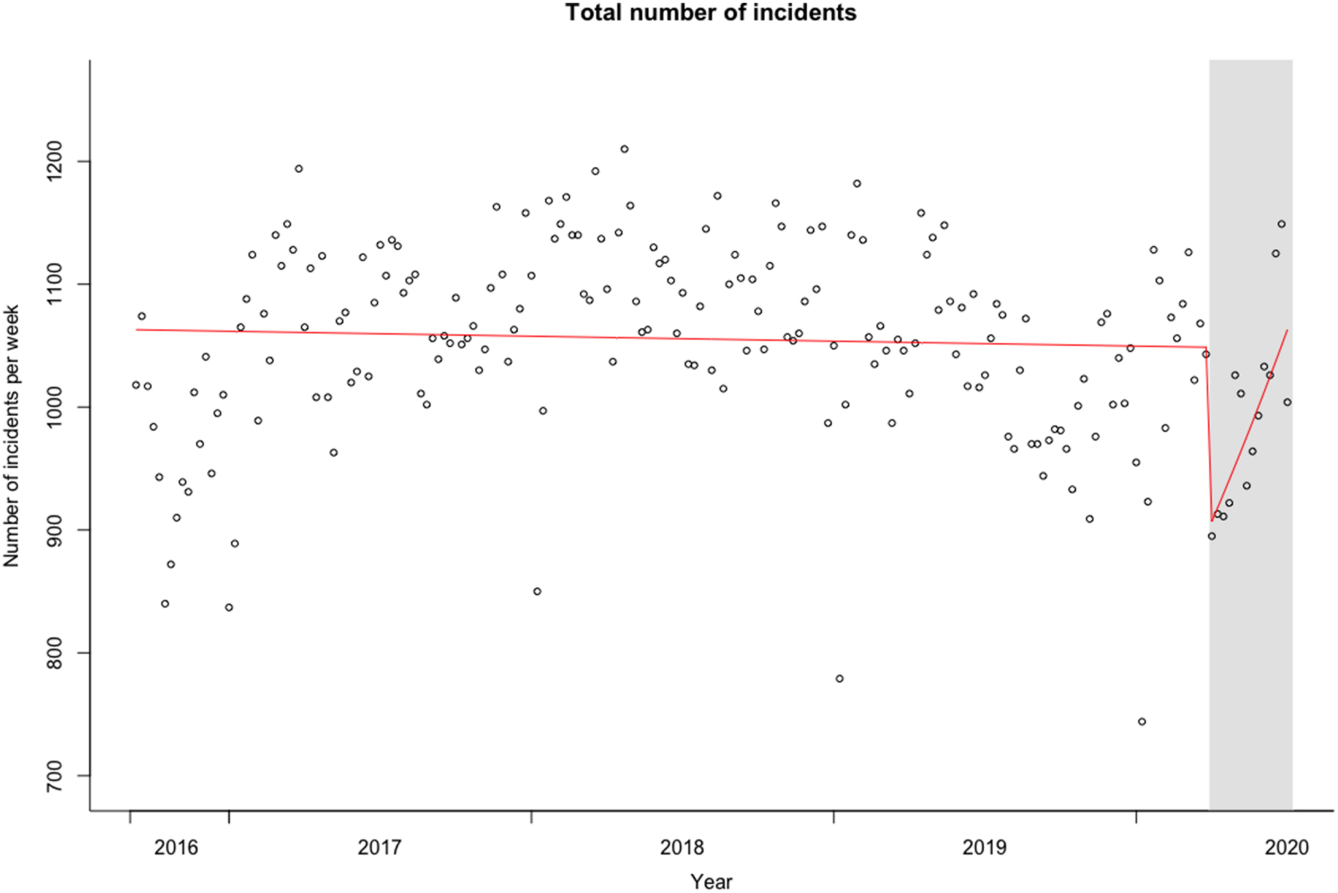
Level of total number of incident reports and seasonality‐corrected estimated regression lines for the pre‐COVID‐19 and COVID‐19 (grey) phases. [Colour figure can be viewed at wileyonlinelibrary.com]

**Figure 2:**
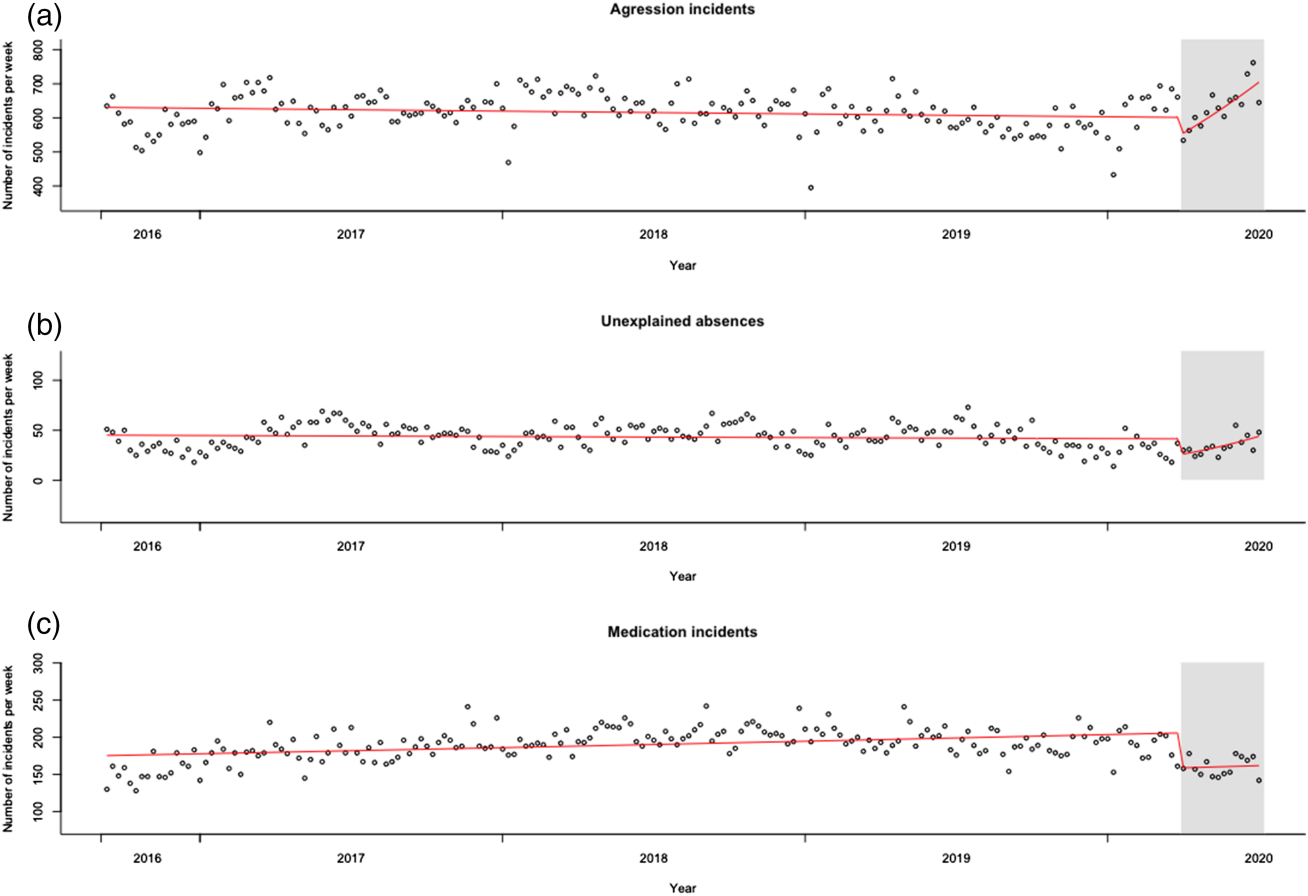
Level of incident reports on aggression (panel a), unexplained absence (panel b), and medication errors (panel c) and seasonality‐corrected estimated regression lines for the pre‐COVID‐19 and COVID‐19 (grey) phases. [Colour figure can be viewed at wileyonlinelibrary.com]

In the first step, metadata were inspected on anomalies and outliers. Outliers resulting from broken consecutive weeks at each year turn were removed by combining these weeks. In the second step, data were detrended using Loess regression and smoothing (Stlplus package; Hafen 2016) and subsequently tested for possible seasonality using a decision rule optimised by Ollech (2019; seastest package) that classifies a time series as seasonal if either the QS‐test value is significant at *P* < .01 or the Kruskall–Wallis test is significant at *P* < .002. Significant seasonal variation was modelled and subtracted from the raw counts to obtain residual values for further analyses. In the third step, change in intercept and slope was tested from the pre‐COVID‐19 phase to the COVID‐19 phase using Poisson regression, following the recommendations by Bernal *et al*. (2016). It should be noted that Poisson regression coefficients cannot be interpreted in a straightforward, linear way like changes per week. For each unit of change in the independent variable, the regression coefficient represents the change in the logarithmic value of expected counts, taking the other independent variables in the prediction into account. Figures 1 and 2 were therefore produced depicting both raw observed and predicted values to be able to gauge the order of magnitude of the effects. In the fourth step, sensitivity tests were conducted by testing change in slopes using the robust Theil–Sen method for computing slopes of regression lines by taking the median of the slopes of all regression lines that may be drawn through the data series (Wilcox 1998). Statistical significance was set at *P* < .05 for the total number of incident reports and medication errors and at *P* < .025 for the subcategories of aggression and unexplained absence. All analyses were performed in r Version 3.6.2 (R Core Team, 2017). The full code and data can be found at https://osf.io/f9mpd/.

## Results

### Total number of incident reports

The metadata provided by 's Heeren Loo spanned *N* = 199 weeks (after combining consecutive broken weeks), for a mean total of 1050.9 incident reports per week (SD = 78.8). Figure 1 shows the observed numbers of total incidents. After detrending the data, seasonality was detected. Analyses were therefore conducted on the residuals of raw counts minus the counts according to the seasonality estimate. Furthermore, data were inspected for a possible change in trend on the gradual introduction of a new system for reporting incidents, but no such trend was seen. Poisson regression analysis of number of incident reports per week on phase showed a significant drop from pre‐COVID‐19 levels to the start of the COVID‐19 phase (*b* = −0.16; *SE* = 0.034; *t* = −4.66; *P* < .001). Figure 1 also shows the slopes for the pre‐COVID‐19 and COVID‐19 phases. While overall the number of incident reports was stable across the years (*b* = −0.00007; *SE* = 0.00008; *t* = −0.97; *P* = .33), the slope for the COVID‐19 period was significantly higher than for the pre‐COVID‐19 period, showing an increasing number of reported incidents (*b* = 0.012; *SE* = 0.0038; *t* = 3.25; *P* = .001).

### Number of reports of aggression and unexplained absence

Across the study period, a mean total of 616.8 incident reports per week (SD = 54.0) regarded aggression and significant seasonality was identified. On average 42.6 incident reports/week (*SD* = 11.8) regarded unexplained absence, while seasonality was not significant. Figure 2a, b shows observed incident counts and the estimated slopes for the pre‐COVID‐19 and COVID‐19 phases. Poisson regression analysis for incidents with aggression showed a significant drop from pre‐COVID‐19 levels to the start of the COVID‐19 phase (*b* = −0.099; *SE* = 0.037; *t* = −2.67; *P* = .008). Aggressive incidents showed a linear decline over the years (*b* = −0.00026; *SE* = 0.00009; *t* = −3.06; *P* = .003). However, the slope for the COVID‐19 period was significantly higher than for the pre‐COVID‐19 period, showing inversion from a negative to a positive trend in incidents with aggression (*b* = 0.019; *SE* = 0.0041; *t* = 4.60; *P* < .001). Poisson regression analysis for unexplained absences showed a significant drop from pre‐COVID‐19 levels to the start of the COVID‐19 phase (*b* = −0.50; *SE* = 0.19; *t* = −2.68; *P* = .008). Unexplained absences were stable over the years (*b* = −0.00047; *SE* = 0.00037; *t* = −1.27; *P* = .20). While the slope for the COVID‐19 period was significantly higher than for the pre‐COVID‐19 period, the increase in unexplained absences during the COVID‐19 period was not significant on the corrected significance level of *P* < .025 (*b* = 0.04; *SE* = 0.020; *t* = 2.02; *P* = .045).

### Number of medication error incidents

Across the study period, a mean of 188.0 incident reports per week (SD = 22.0) regarded medication errors. No seasonality was identified. Figure 2c shows observed incident counts and the estimated slopes for the pre‐COVID‐19 and COVID‐19 phases. Poisson regression analysis showed a significant drop in medication error reports from pre‐COVID‐19 levels to the start of the COVID‐19 phase (*b* = −0.26; *SE* = 0.064; *t* = −4.09; *P* < .001). Medication error reports increased over the years (*b* = 0.00087; *SE* = 0.00014; *t* = 6.29; *P* < .001). The slopes for the COVID‐19 period and the pre‐COVID‐19 period were not significantly different (*b* = 0.0005; *SE* = 0.0071; *t* = 0.07; *P* = .94).

### Sensitivity tests

Testing the change in slopes from the pre‐COVID‐19 to COVID‐19 period on the deseasonalised residuals using the robust Theil–Sen test was consistent with the Poisson regressions using Bernal *et al*.'s (2016) method for total incidents (ΔTheil–Sen = 12.80; *SE* = 4.72; *P* = .007), for incidents with aggression (ΔTheil–Sen = 11.46; *SE* = 3.49; *P* = .001), and for medication errors (ΔTheil–Sen = 0.16; *SE* = 0.03; *P* = 1.0). For unexplained absences, in contrast to the Poisson regression, the Theil–Sen method showed a significant increase in slope (ΔTheil–Sen = 1.44; *SE* = 0.59; *P* = .001).

## Discussion

The drastic measures taken by authorities against COVID‐19 coincided with changes in weekly incident reports of a large care organisation supporting people with intellectual disability in the Netherlands. Drops in incident reports could be observed immediately after these measures were announced and implemented, but this went along with an unexpected drop in medication error reports, which leaves open the possibility that workload and pressure on direct care staff led to a lower rate of incident reporting. However, while incidents with medication errors remained stable, increases were observed for other incidents, in particular incidents that involved aggression.

It is important to note that the increasing number of incidents in the COVID‐19 phase remained within the bounds observed during the preceding period. Continued monitoring should reveal whether the rise in incidents flattened after this initial response phase, thanks to scaling down of many of the countermeasures against the pandemic. Nevertheless, some of the countermeasures were kept in place, such as keeping of 1.5‐m distance, and clinical services, education, training, sports and work were not fully restored. Furthermore, the greater differentiation in countermeasures might be more difficult to understand and comply with, providing new grounds for conflict. A continuation of the rise in aggressive incidents would be especially concerning because these incidents are highly stressful for clients and staff and may be especially stressful in combination with risk of infection. It would also be important to monitor the lower rate of medication error incidents. Lower rates of reporting might contribute to health risks. However, it is also conceivable that the COVID‐19‐countermeasures have increased compliance with health care protocols within more structured day routines, possibly leading to an actual decrease in errors with taking medicine.

While the use of administrative data made this rapid assessment of potential impact of COVID‐19 possible, the limitations of these data should be kept in mind. Data may not only be uncertain due to potential changes in tendency to file incident reports, but the lack of data on the nature of the incidents, clients and settings means that the current findings are merely a distal reflection of the dynamics of adapting to a system wide challenge such as a pandemic. It should be noted that the organisation showed a steady rise in number of clients over the years that was not accounted for in the rate of incidents, which can be explained by the number of clients living in the care homes of the organisation remaining constant and they likely account for almost all incidents. Findings from administrative data may be most useful for starting conversations with clients and staff close to the source of these data (Wolpert and Rutter 2018), helping to put their unique experiences and challenges during this pandemic (e.g., Embregts *et al*. in press‐a, b) on the agenda and into a wider perspective. Clients have been found to attribute challenging behaviours to a range of situational and social factors (Van den Bogaard *et al*. 2019). Their views on the rise in incidents with aggression in response to drastic changes in their situation might therefore be especially informative for understanding the impact of COVID‐19 countermeasures. The current findings underline calls for an inclusive approach to taking sustainable measures to control COVID‐19 (Berger *et al*. 2020) or other pandemics in the future.

## Source of funding

Part of this work was made possible by the program for Academic Collaborative Centers for Intellectual Disability of ZonMW.

## Conflict of interest

No conflicts of interest have been declared.
